# Do population-level risk prediction models that use routinely collected health data reliably predict individual risks?

**DOI:** 10.1038/s41598-019-47712-5

**Published:** 2019-08-02

**Authors:** Yan Li, Matthew Sperrin, Miguel Belmonte, Alexander Pate, Darren M. Ashcroft, Tjeerd Pieter van Staa

**Affiliations:** 1 Health e-Research Centre, School of Health Sciences, Faculty of Biology, Medicine and Health, The University of Manchester, Manchester Academic Health Sciences Centre (MAHSC), Oxford Road, Manchester M13 9PL UK; 2 Centre for Pharmacoepidemiology and Drug Safety, School of Health Sciences, Faculty of Biology, Medicine and Health, University of Manchester, Oxford Road, Manchester M13 9PL UK; 3 NIHR Greater Manchester Patient Safety Translational Research Centre, School of Health Sciences, Faculty of Biology, Medicine and Health, University of Manchester, Oxford Road, Manchester M13 9PL UK; 4 Utrecht Institute for Pharmaceutical Sciences, Utrecht University, Utrecht, Netherlands; 5 Alan Turing Institute, Headquartered at the British Library, London, UK

**Keywords:** Epidemiology, Epidemiology

## Abstract

The objective of this study was to assess the reliability of individual risk predictions based on routinely collected data considering the heterogeneity between clinical sites in data and populations. Cardiovascular disease (CVD) risk prediction with QRISK3 was used as exemplar. The study included 3.6 million patients in 392 sites from the Clinical Practice Research Datalink. Cox models with QRISK3 predictors and a frailty (random effect) term for each site were used to incorporate unmeasured site variability. There was considerable variation in data recording between general practices (missingness of body mass index ranged from 18.7% to 60.1%). Incidence rates varied considerably between practices (from 0.4 to 1.3 CVD events per 100 patient-years). Individual CVD risk predictions with the random effect model were inconsistent with the QRISK3 predictions. For patients with QRISK3 predicted risk of 10%, the 95% range of predicted risks were between 7.2% and 13.7% with the random effects model. Random variability only explained a small part of this. The random effects model was equivalent to QRISK3 for discrimination and calibration. Risk prediction models based on routinely collected health data perform well for populations but with great uncertainty for individuals. Clinicians and patients need to understand this uncertainty.

## Introduction

Cardiovascular disease (CVD) was the primary cause of death in USA, Europe and China in 2017^1^. Multiple studies have suggested that the identification of patients with high CVD risk is important in its prevention^2–5^. Risk prediction models are often used to predict CVD risk for individual patients^5^. Examples are the Framingham risk score (FRS) and QRISK which provide risks of developing CVD in the next 10 years. Information is used on risk factors such as age, gender, body mass index (BMI), ethnicity, smoking history and disease histories^6,7^. FRS models have good performance in the USA population, but the risk predictions may be problematic when applied to cohorts that are hugely different from the cohort used for model development^8^. In the UK, treatment guidelines for the primary prevention of CVD recommend the use of QRISK2 (second version) to identify patients with high CVD risk^9^.

QRISK is based on routinely collected data from general practices in the UK^7^. Conventional approaches were used to measure discrimination and calibration in the overall population^7^. However, there can be substantial variation between general practices in the style of coding clinical information (coding style) and completeness of data recording^10^. Different coding dictionaries are also currently being used in UK primary care as the EHR systems either use Read version 2 or CTV3 codes^11^. The patient case-mix (referring to a variation in risk factors for disease) may also vary between practices. This variability in the underlying data sources is currently not routinely considered in the development of risk prediction models, but it could potentially lead to heterogeneity in the prediction model’s performance^12^. The objective of this study was to assess the level of generalisability of risk prediction models that are based on routinely collected data from EHRs, and to measure the effects of practice heterogeneity on the individual predictions of risk. The QRISK3 prediction model (for the 10 year risk of CVD) was used as an exemplar.

## Methods

### Data source

This study used data from the Clinical Practice Research Datalink (CPRD) which is a database with anonymised EHRs from 674 GP practices in the UK. The database includes 4.4 million (6.9% of the UK population) patients and is broadly representative of the UK general population in terms of age, gender and ethnicity^13^. CPRD includes patient records of demographics, symptoms, tests, diagnoses, therapies, health-related behaviours and referrals to secondary care. Data from over half of the practices have been linked using unique patient identifiers to other datasets from secondary care, disease-specific cohorts and mortality records^13^. This study was restricted to 392 general practices that have been linked to Hospital Episode Statistics (HES), Office for National Statistics (ONS) and Townsend scores^7^. Over 1,700 publications have used CPRD data^14^. Previously, CPRD data has been used to externally validate QRISK2^15^.

### QRISK prediction models

QRISK is a statistical model which is being used to predict a patient’s risk over 10 years of developing CVD (including coronary heart disease, stroke or transient ischaemic attack). The second version (QRISK2) was derived in 2008 using data from 355 practices in the QResearch database^16^, and validated using data from 364 practices from the THIN database^17^. QRISK3 is the latest version published in 2017, which includes more clinical variables, such as migraine and chronic kidney disease, than QRISK2^7^. The QRISK3 predicted risks were calculated using the open access algorithm^18^. Calculations were successfully verified to be the same as predictions by the online calculator. This was done for simulated different patient groups in which each risk factor was changed sequentially covering the changes of all QRISK3 risk factors.

### Study population

The study population in this study was similar to that used for the development cohort for QRISK3^7^. Patients were included if they were aged between 25 and 84 years, had no CVD history or prescribing of statins prior to the index date. The follow-up of patients in CPRD cohort started one year after start of data collection, patient’s registration date, date of reaching age 25 years, or January 1 1998 (whatever came last) and it ended at the end of data collection, a patient leaving the practice, date patient’s death or the CVD outcome (whatever came first). Patients were censored by the earliest date among the first statin prescription, transfer or the end of follow-up^19^. The index date (as the start date for evaluating CVD and the baseline date for assessing a patient’s history) was chosen randomly from the period of follow-up. The random index date^19^ was preferred, because it gets a better spread of calendar time and age, and captures the time-relevant practice variability (e.g., change of recording and second trend of CVD incidence rate). This study considered the same risk factors as in QRISK3^7^.

### Statistical analysis

The QRISK3 predicted risks were estimated for each patient and were also averaged within each practice. Averaged predicted risks were compared to the observed risks at year 10 which were based on Kaplan Meier life tables. The observed risks were extrapolated for the 13.5% of practices with less than 10 years of follow-up. It was assumed that the life tables of these practices followed the pattern of the overall population life table. We calculated each year’s CVD relative risk (RR) by dividing the current year’s CVD proportion by the next year’s CVD proportion. The extrapolation was verified using practices with 10 years follow-up. Specifically, we randomly remove records to make these practices have less than 10 years follow-up and then compared the extrapolated risk to the observed risk. We found no evidence^20^ that the extrapolated risks were statistically significant to the actual observed risks.

A Cox model with a frailty (random effect) term for each practice was fitted to assess the effects of practice heterogeneity^21^. Patient survival time (time until censoring or CVD) was the outcome (dependent variable) and the linear predictor from the QRISK3 model was included as an offset. Each patient’s linear predictor was calculated using the patient’s risk factors and corresponding QRISK3 coefficients. Each practice’s random effects on individual risk prediction and the standard deviation of all practices’ random effects were extracted from the frailty model. Patient QRISK3 predictions and their corresponding practice random effects were combined to calculate a random effects model predicted risk. These were compared with the QRISK3 predicted risks. The distribution of the differences between the QRISK3 and the random effects model’s predicted risks were plotted.

Limited practice size or duration of follow-up could contribute to the unknown variability between risks predicted by QRISK3 and the random effects model. In order to measure this random error, we simulated data under a null hypothesis of no practice level variability and estimated the distribution of the practice level random effects, and compared this with the distribution of the practice level random effects observed in the CPRD data (i.e. a permutation test). Specifically, simulations were conducted using 2,000 datasets of the same size and follow-up as the CPRD data. The CVD outcomes were simulated by assigning a random probability from a uniform distribution (0, 1) to each patient. The random effects model was then fitted to these simulated data in order to quantify the random variability. The comparison between effects of unknown random variability and effects of practice level variability on individual patients was plotted using one million patients (50% male and 50% female) who had a QRISK3 predicted risk of 10%.

We used classical model performance measurements to compare QRISK3 with the random effects model. The data from each practice were randomly divided into two (70% and 30%) stratified by gender. The first part was used to develop the random effects model and the second part to test and calculate model performance measurements including the C-statistic^22^, brier score^23,24^ and net benefit^25^. These measurements were calculated using QRISK3 predictions, predictions of random effects model, patient follow-up time and patient status at the time of censoring. Empirical confidence intervals were calculated using 1,000 bootstrap samples.

Missing values for ethnicity, BMI, Townsend score, systolic blood pressure (SBP), standard deviation of SBP, cholesterol, High-Density Lipoprotein (HDL) and smoking status (only these have missing values) were imputed using Markov chain Monte Carlo (MCMC) method with monotone style^26^. The QRISK3 and random effects risks were then averaged based on ten imputations. We calculated random effects of CPRD practices and random effects separately for females and males consistent with QRISK3 development. The random effects of practices were calculated independently by both SAS and R with almost identical results. The random effects model used procedures from SAS 9.4 and “coxme” package for the R 3.4.2. The analyses of the datasets, missing value imputation, extrapolation validation and life tables were produced by SAS. R was used to model the data. The protocol for this work was approved by the independent scientific advisory committee for Clinical Practice Research Datalink research (protocol No 17_125RMn2). We confirm that all methods were performed in accordance with the relevant guidelines and regulations.

## Results

Table 1 shows the patient characteristics and level of data recording across the 392 general practices. The mean age of patients varied between practices (5% percentile was 40.0 years and 95% percentile was 49.8 years). Presence of CVD risk factors also varied between practices. The 5–95% range between practices was 1.9 to 16.4 for recorded history of severe mental illness. The level of data completeness also varied substantially between practices. Ethnicity was not recorded for 19.6% of patients in the 5^th^ percentile of practices compared to 93.9% in the 95% percentiles. Life table analysis are shown in eTable 1 in the Supplement.

**Table 1:** Characteristics of the general practices included in the study and the distribution of data recording.

	Mean (SD)	Distribution of characteristics across practices: Percentiles				
		5^th^	25^th^	50^th^	75^th^	95^th^
**General characteristics of practices**						
Total number of CVD events over 10 years in each practice	266.4 (176.5)	17.0	129.5	251.5	376.5	581.0
Average age of patients in each practice	44.9 (3.0)	40.0	42.9	45.0	46.7	49.8
% female patients	51.2 (2.1)	47.5	50.1	51.2	52.4	54.5
Total number of patients in each practice	9262.3 (5072.9)	2305.0	5292.5	8792.5	12180.0	17616.0
**CVD risk factors**						
% patients with alcohol abuse	1.4 (1.2)	0.5	0.8	1.1	1.6	3.0
% patients with anxiety	13.8 (5.3)	6.5	10.0	13.1	16.9	23.4
% patients with HIV	0.1 (0.1)	0.0	0.0	0.1	0.1	0.3
% patients with left ventricular hypertrophy	0.2 (0.1)	0.1	0.1	0.2	0.3	0.5
% patients with atrial fibrillation	0.7 (0.3)	0.3	0.5	0.7	0.9	1.3
% patients on atypical antipsychotic medication	0.4 (0.2)	0.2	0.3	0.4	0.6	0.9
% patients with Chronic kidney disease (stage 3, 4 or 5)	1.0 (0.9)	0.3	0.6	0.9	1.3	2.1
% patients on regular steroid tablets	0.1 (0.1)	0.0	0.0	0.1	0.1	0.2
% patients with erectile dysfunction	1.5 (0.6)	0.7	1.1	1.5	1.8	2.4
% patients with angina or heart attack in a 1st degree relative <60	3.6 (3.0)	0.7	1.8	2.9	4.4	8.7
% patients on blood pressure treatment	6.8 (1.9)	3.8	5.6	6.7	8.2	9.9
% patients with migraines	6.4 (2.1)	3.2	4.8	6.4	7.8	9.6
% patients with rheumatoid arthritis	0.6 (0.2)	0.3	0.5	0.6	0.7	1.0
% patients with severe mental illness (this includes schizophrenia, bipolar disorder and moderate/severe depression)	7.8 (4.5)	1.9	4.2	7.2	10.8	16.4
% patients with Systemic Lupus Erythematosus	0.1 (0.0)	0.0	0.0	0.1	0.1	0.1
**SBP**						
Average SBP within practice	126.8 (2.8)	122.3	125.1	126.8	128.8	131.0
% patients with missing SBP	25.5 (7.3)	13.9	20.7	25.3	30.0	38.5
Average SBP standard deviation within practice	9.9 (0.7)	8.9	9.5	9.9	10.3	11.0
% patients with missing SBP standard deviation	52.7 (7.7)	39.0	48.3	53.1	57.3	64.7
**BMI**						
Average BMI when recorded	26.4 (0.7)	25.0	25.9	26.4	26.9	27.5
% patients with missing BMI	39.2 (11.8)	18.7	31.2	39.1	46.6	60.1
**Cholesterol/HDL ratio**						
Average Cholesterol/HDL ratio	4.0 (0.2)	3.6	3.8	4.0	4.1	4.4
% patients with missing Cholesterol/HDL ratio	64.4 (10.0)	48.2	57.6	63.9	70.4	81.6
**Smoking**						
% patients who never smoked	47.8 (7.6)	36.0	43.3	47.9	52.7	59.4
% ex-smokers	22.3 (5.2)	13.8	19.0	22.5	25.5	30.9
% current-smokers	29.8 (7.0)	19.9	25.1	29.2	33.8	42.7
% patients with missing smoking status	24.2 (8.6)	10.3	18.6	23.8	29.5	39.4
**Diabetes**						
% patients with type 1 diabetes	0.2 (0.1)	0.1	0.2	0.2	0.3	0.4
% patients with type 2 diabetes	1.3 (0.4)	0.6	1.0	1.3	1.6	2.0
Ethnicity						
% other Asian patients	1.9 (3.2)	0.0	0.3	0.9	1.9	7.6
% Bangladeshi patients	0.4 (1.3)	0.0	0.0	0.2	0.4	1.4
% Black patients	3.5 (5.9)	0.1	0.5	1.3	3.4	15.3
% Chinese patients	0.7 (0.7)	0.0	0.2	0.5	1.0	2.0
% Indian patients	2.7 (5.3)	0.0	0.3	1.1	2.9	10.6
% patients with other ethnicity	2.9 (3.0)	0.3	0.9	2.0	3.6	9.1
% Pakistani patients	1.2 (3.6)	0.0	0.1	0.3	0.9	4.7
% White patients	86.7 (15.5)	48.2	83.4	92.3	96.8	98.8
% patients with missing ethnicity	58.5 (23.7)	19.6	38.5	62.5	77.5	93.9
**Townsend score (Socioeconomic Status)**						
% patients with Townsend score 1 (the least deprived)	20.3 (19.2)	0.1	4.1	14.7	31.1	59.7
% patients with Townsend score 2 (less deprived)	21.3 (16.4)	0.6	8.8	18.6	30.3	51.8
% patients with Townsend score 3 (deprived)	21.2 (13.1)	2.4	12.1	18.5	29.4	44.8
% patients with Townsend score 4 (more deprived)	21.1 (15.5)	0.3	8.6	19.9	29.5	52.9
% patients with Townsend score 5 (the most deprived)	16.1 (21.8)	0.0	0.4	7.6	22.3	66.3
% patients with Townsend score missing	0.1 (0.6)	0.0	0.0	0.1	0.1	0.3

**Figure 1:**
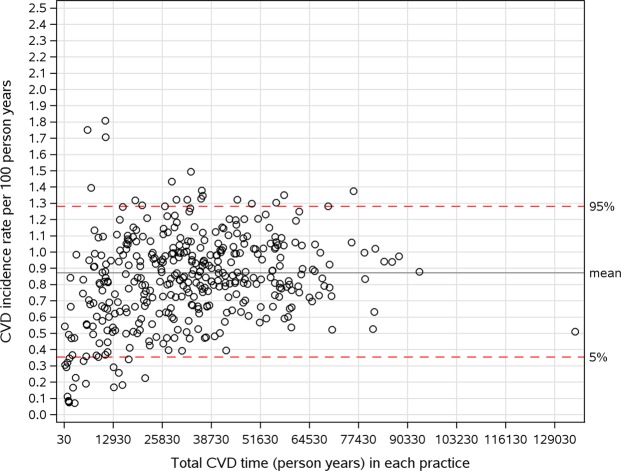
Variation of CVD incidence rate (per 100 person years) across practice.

Figure 1 shows the variation of CVD incidence rate among practices by plotting CVD incidence rate per 100 person years against the total follow-up time. A large amount of variation of CVD incidence rate were found between practices.

**Figure 2:**
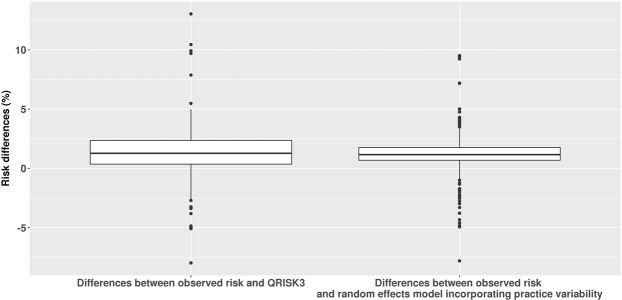
Comparison of differences between observed and QRISK3 (random effects) mode.

Figure 2 shows that the random effects model has less variation of differences between observed and predicted risk on practice level than QRISK3.

Random effects model’s Brier score (0.067 (95% CI: 0.0667, 0.0682)) was close to QRISK3’s brier score (0.067 (95% CI: 0.0666, 0.0680)). The difference of Brier score between random effects model and QRISK3 was 0.002 (95% CI: 0.00008, 0.0023). Random effects model’s C-statistic (0.852 (95% CI: 0.850, 0.854)) was also close to QRISK3’s C-statistic (0.850 (95% CI: 0.848, 0.852)). The difference of C-statistic between the two models was 0.0017 (95% CI: 0.0015, 0.0020). The net benefit analysis^25^ shows that both of models could predict three true CVD events without adding a false negative CVD events in every 100 patients with a given threshold of 10% (visualised in eFigure 2 in the Supplement). Standard deviation of random effects of CPRD practice between females (0.174) and males (0.177) were close to each other.

Table 2 shows the inconsistencies between the risks predicted for the same group of individual patients by QRISK3 and the random effects model (visualised in eFigure 1 in the Supplement). Patients with a predicted QRISK3 risk between 9.5~10.5% were found to have a much larger range of risks in the random effects model (between about 7.6~13.3%). Table 2 also shows the level of reclassification to below or above the treatment risk threshold of 10% when using the random effect model instead of the QRISK3 predicted risk. It was found that 19.7% patients with QRISK3 predicted risk between 8.5~9.5% had a risk above the treatment threshold when using the random effects model. For patients with QRISK3 predicted score between 10.5~11.5%, 24.4% of patients were reclassified to below the treatment threshold when using the different model.

**Table 2:** Inconsistencies between individual CVD risks as predicted by QRISK3 or by random effects model that incorporated practice variability.

QRISK3 predicted CVD risk(over 10 years)	Predicted risk according to random effects model incorporating practice variability							Total number of patients
	Percentile					% below/above treatment threshold of 10 year CVD risk (10%)		
	2.5^th^~97.5^th^	5^th^	25^th^	75^th^	95^th^	≤10	>10	
<6.5	0.1~6.0	0.1	0.4	2.6	5.4	100.0	0.0	2561602
6.5~7.5	5.3~9.4	5.5	6.3	7.6	8.9	99.0	1.0	96981
7.5~8.5	6.0~10.7	6.3	7.2	8.7	10.2	94.0	6.0	82768
8.5~9.5	6.8~12.0	7.1	8.2	9.7	11.4	80.3	19.7	72098
9.5~10.5	7.6~13.3	7.9	9.1	10.8	12.6	54.0	46.0	64477
10.5~11.5	8.4~14.6	8.8	10.0	11.9	13.9	24.4	75.6	56550
11.5~12.5	9.2~15.8	9.6	11.0	13.0	15.1	9.1	90.9	50278
12.5~13.5	10.0~17.1	10.4	11.9	14.0	16.3	2.4	97.6	45126
≥13.5	12.7~55.4	13.5	17.8	34.7	50.2	0.1	99.9	600938

**Figure 3:**
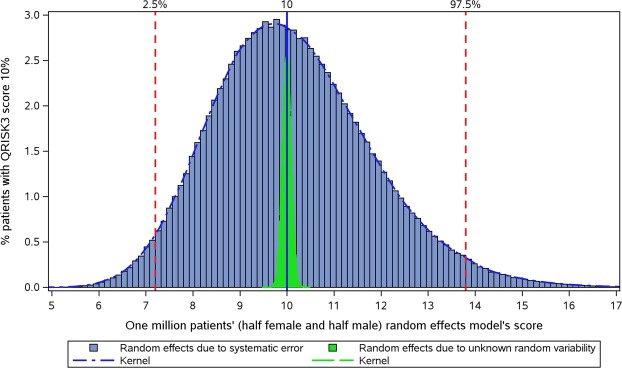
Distribution of predicted risks in the random effects model for patients with a QRISK3 predicted risk of 10% (using simulations in order to estimate the extent of random variability).

Figure 3 plots the distribution of risks predicted with the random effect model for those with a QRISK3 predicted risk of 10%. The effects of random variability (measured by simulation analysis) in the random effect model is also presented in this figure. It was found that the effect of practice variability on predicted risks for patients cannot be fully explained by random variability, as the overall distribution (blue area) with a random effects’ standard deviation of about 0.17 was much larger than the distribution due to random variability (green area) with a standard deviation for random effects of about 0.01.

## Discussion

### Key results

This study found that incorporating practice variability in a risk prediction model substantially affected the predicted CVD risks of individual patients. The random effect model was similar to QRISK3 in terms of calibration and discrimination. Patients with a QRISK3 predicted risk of 10% had a much larger range of predicted risks after incorporating practice variability. Treatment classifications were found to be different for a substantive number of patients after considering the heterogeneity in CVD incidence between practices.

### Limitation

There are several limitations of this study. Firstly, the observed risks had to be extrapolated for the practices with less than 10 years of follow-up to compare with QRISK3 (or random effects model) on practice level. The QRISK3 developers did not share the life table pattern of CVD risks over follow-up in QResearch. Although the validation showed that the result of extrapolation was not statistically significantly different from those practices with 10 years follow-up, the use of the actual changes in CVD risk over 10 years would have been preferable. Also, the definitions and classification of the risk factors could have been different from QRISK3 as the underlying EHR software systems vary between CPRD and QResearch (Vision and EMIS, respectively). However, the calibration and discrimination of QRISK3 in CPRD were consistent with those reported for QResearch, which suggest that the effects of differences in definitions was minimal.

### Interpretation

Risk prediction models need to provide accurate and generalisable predictions in order to be used clinically for individual patient decision making^27^. Current guidelines for the development of risk prediction models do not include the evaluation of extent of heterogeneity in the underlying population (unaccounted for by the model) and its impact on the generalisability of the model. Conventional metrics in the evaluation of risk prediction models only include population level averages such as calibration and discrimination^28^. However, literature suggests that the risks at the population and individual levels may be determined differently^29,30^. An example of a tool with an acceptable average measurement but unacceptable generalisability due to heterogeneity would be a blood pressure measurement that has systematic measurement errors at different times of a day. The historic treatise by Rose emphasised that the ability to predict an average risk on a population level does not always equate to the prediction of the individuals who are going to have the event soon^31^. A previous study highlighted that the Framingham and QRISK2 risk prediction models showed considerable variability in predicting high CVD risk despite comparable population-level calibration and discrimination^19^. As Briggs emphasised, risk prediction models that provide non-extreme probabilities can never empirically be proven wrong. It was also suggested, as done in the present study, to compare the impact on predictions and decision-making with different models that are statistically comparable^32^. Our study found that, the predicted CVD risks for individuals were very different after incorporating previously unmeasured variability between practices and that decisions based on the QRISK3 or random effect model could be quite different.

There may be several reasons for our finding of heterogeneity between general practices unaccounted for by QRISK3. One reason may be that the data quality of EHRs varies between general practices. A study on the EHR recording of osteoporosis reported that there was variability in inter-practice data quality with clinically important codes and with multiple ways that the same clinical concept was represented^33^. Also, different practice computer systems have different versions of clinical coding^33^. Damen *et al*. in their recent literature review of all CVD prediction models, pointed out that consistent codes such as ICD-9 or ICD-10 should be used in models’ development and validation, as different definitions of CVD outcome lead to variation of model performance^5^. Another reason may be unmeasured heterogeneity in CVD risks in the populations of the different practices. There is substantive evidence that risks of disease are not uniformly distributed. A nation-wide study reported that there are severe inequalities in all-cause mortality between the North and South of England from 1965 to 2008^34^. A study by Langford *et al*. reported that region accounted for four times more variation in mortality than that explained by the classification of residential neighbourhoods by household type including socioeconomic status^35^. In order to use a risk prediction model for individual decision making, it should be established whether or not to allow these models to miss important causal predictors. If they do, this can then lead to a substantial misclassification on an individual level.

Riley *et al*. have proposed a statistical way to measure heterogeneity between sites by evaluating the C-statistics across practices in funnel plots with approximate 95% confidence interval based on the observed standard error observed^36^. We replicated Riley’s funnel plot of QRISK2 and found similar variation of the C-statistic among practices in CPRD with QRISK3 (eFigure 4 in the Supplement). But this approach of funnel plots is limited as it does not assess the impact of heterogeneity on individual risk predictions. Random effects models are the standard approach to assess the effects of practice heterogeneity^21^. Our results highlight that it is not enough to only consider calibration and discrimination on the population level when assessing a prediction model’s clinical utility on individual patients. The extent of heterogeneity in risk prediction unaccounted for by the model will need to be evaluated in addition to calibration and discrimination.

### Implications for Research and Practice

This study found that QRISK3 has limited generalisability and accuracy in predicting individual risks in heterogeneous settings. The predictions of CVD risks of individual patients substantially changed after incorporating practice variability which could impact the clinical decisions for many patients. In order to improve the clinical utility of these risk prediction models, the level of unexplained heterogeneity in populations, disease incidence and data quality must be assessed before implementing such models for individual clinical decision making. Given the uncertainty with risk prediction models that use routinely collected EHR data, it is questionable whether these tools should be used without additional clinical interpretation and without incorporating causal risk factors that better capture the unmeasured heterogeneity between different general practices. Recently an online calculator was launched by Public Health England which allows members of the public to estimate their heart age based on a QRISK model^37^. Our study indicates that these estimates could be quite different when incorporating unmeasured heterogeneity and that the level of uncertainty with these predictions is considerable.

## Supplementary information

Supplementary Online Content
