# Supplementary material for: Do population-level risk prediction models that use routinely collected health data reliably predict individual risks?
Source: Sci Rep. 2019 Aug 2;9:11222. doi: 10.1038/s41598-019-47712-5 (PMC6677736; doi:10.1038/s41598-019-47712-5)
Supplement: Supplementary file 1 — Supplementary Online Content [file 41598_2019_47712_MOESM1_ESM.docx]

**Supplementary Online Content**

Do population-level risk prediction models that use routinely collected health data reliably predict individual risks?

Scientific Reports XXX. Published online XXX

Doi: XXXX

**eAppendix.** Interpretation of appendix tables and figures.

**eTable 1.** Distribution across practices of the number of CVD cases, number of patients at risk and survival rate over 10 years

**eFigure 1**. Comparison of random effects model’s score and QRISK3 score

in the same group of patients (grouped by certain range (red lines) of QRISK3 score)

**eFigure 2.** Comparison of net benefit between QRISK3 and random effects model

**eFigure 3-1.** Calibration plot of QRISK3

**eFigure 3-2.** Calibration plot of random effects model

**eFigure 4**. Variation of QRISK3’s C-statistic among practices—a replication of Riley’s funnel plot

**eAppendix.** Interpretation of appendix tables and figures.

[eTable 1](#eTable1) shows the distribution of number of CVD events, number of patients at risk and survival rate among practices at 10 years. The number of CVD events, number of patients at risk and survival rate of practices are generally decreasing along the 10 years. The number of CVD events and the number of patients at risk varied between 5~95% percentile practices. The survival rate of 5~95% practices has less variation. Practices which do not have survival rate in 10 years are because their longest follow-ups are less than 10 years.

[eFigure 1](#eFigure1) visualizes the inconsistencies between the risks predicted for the same group of individual patients by QRISK3 and the random effects model. Patients with a predicted QRISK3 risk between 9.5% ~ 10.5% were found to have a much larger range of risks in the random effects model (between about 6% ~15%)

[eFigure 2](#eFigure2) shows two models’ net benefit is about 3.1% at the threshold 10%, which means both of models predict about 3 true positive CVD events without adding new false positive CVD patients.

[eFigure 3-1](#eFigure31) and [3-2](#eFigure32) show two models have similar calibration.

[eFigure 4](#eFigure3) is a replication of Riley’s ^1^ funnel plot. The left panel shows that QRISK3 has variation of C-statistic among practices, and the right panel performed a formal meta-analysis to identify outlier practices (those red dots outside the 95% prediction interval). The figure shows that QRISK3 performs differently on different practices, which is consistent to Riley’s^1^ finding on QRISK2.

**References to online-only supplement**

1. Riley, R. D. *et al.* External validation of clinical prediction models using big datasets from e-health records or IPD meta-analysis: opportunities and challenges. *BMJ* **353,** i3140 (2016).

**eTable 1. Distribution across practices of the number of CVD cases, number of patients at risk and survival rate over 10 years**

|  | **Number of patients with CVD events in practices (Percentile)** | | | | **Number of patients at risk^a^ in practices (Percentile)** | | | | **Life table estimate of proportion of patients without CVD (Percentile)** | | | |
| --- | --- | --- | --- | --- | --- | --- | --- | --- | --- | --- | --- | --- |
| **Year** | **5^th^** | **25^th^** | **75^th^** | **95^th^** | **5^th^** | **25^th^** | **75^th^** | **95^th^** | **5^th^** | **25^th^** | **75^th^** | **95^th^** |
| 1 | 5.0 | 31.0 | 83.5 | 134.0 | 1789.0 | 4358.0 | 10247.5 | 14843.0 | 1.00 | 1.00 | 1.00 | 1.00 |
| 2 | 2.0 | 21.0 | 60.0 | 93.0 | 575.0 | 3019.3 | 7352.0 | 10560.0 | 0.99 | 0.99 | 0.99 | 1.00 |
| 3 | 0.0 | 14.0 | 49.0 | 76.0 | 85.5 | 2324.8 | 5763.3 | 8329.0 | 0.97 | 0.98 | 0.99 | 0.99 |
| 4 | 0.0 | 12.0 | 41.0 | 64.0 | 0.0 | 1821.3 | 4671.0 | 6707.5 | 0.95 | 0.97 | 0.98 | 0.99 |
| 5 | 0.0 | 10.5 | 34.0 | 54.0 | 0.0 | 1456.0 | 3893.0 | 5530.5 | NA^b^ | 0.96 | 0.98 | 0.98 |
| 6 | 0.0 | 9.0 | 29.5 | 48.0 | 0.0 | 1128.8 | 3225.0 | 4713.5 | NA | 0.95 | 0.97 | 0.98 |
| 7 | 0.0 | 7.0 | 26.0 | 42.0 | 0.0 | 839.0 | 2688.5 | 3995.5 | NA | 0.94 | 0.96 | 0.97 |
| 8 | 0.0 | 5.0 | 22.0 | 39.0 | 0.0 | 601.3 | 2208.0 | 3345.0 | NA | 0.93 | 0.95 | 0.97 |
| 9 | 0.0 | 4.0 | 18.0 | 32.0 | 0.0 | 420.0 | 1803.8 | 2788.0 | NA | 0.92 | 0.94 | 0.96 |
| 10 | 0.0 | 2.0 | 15.0 | 26.0 | 0.0 | 263.8 | 1426.8 | 2297.5 | NA | 0.91 | 0.93 | 0.95 |
| **a. Number of patients in the middle point of each year was used  b. NA is because practices have less than 10 years follow-up data** | | | | | | | | | | | | |


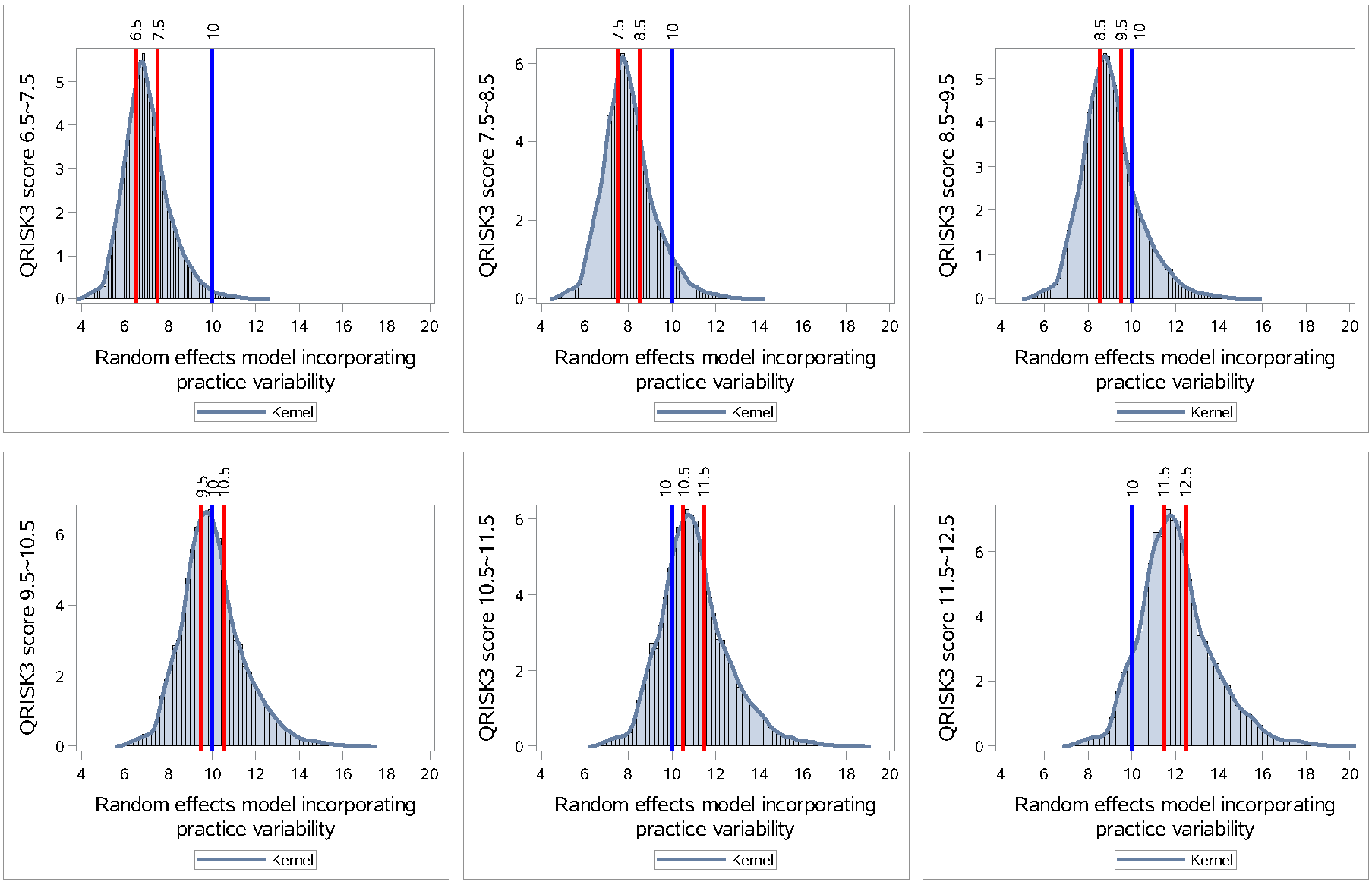


**eFigure 1. Comparison of random effects model’s score and QRISK3 score**

**in the same group of patients (grouped by certain range (red lines) of QRISK3 score)**

**
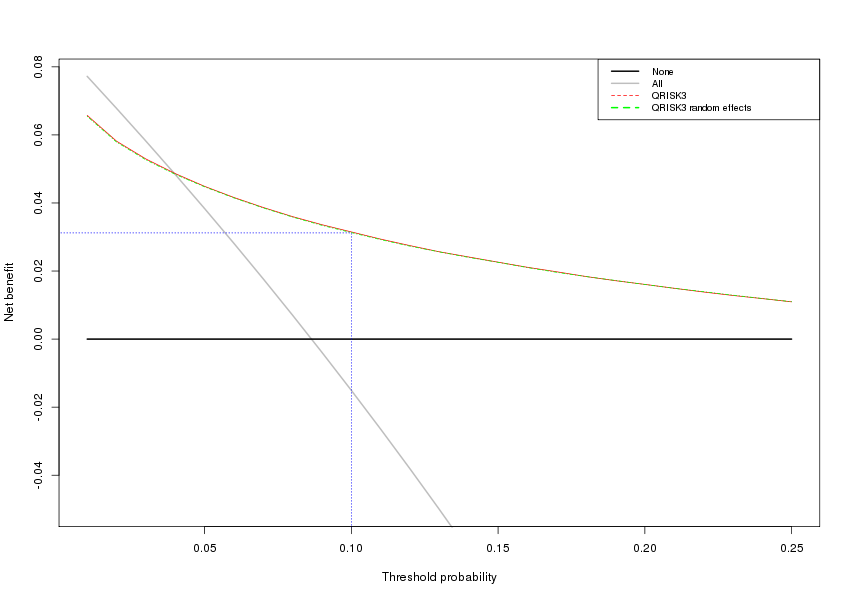
**

**eFigure 2. Net benefit analysis on QRISK3 and random effects model**

**eFigure 3-1. Calibration plot of QRISK3**

**eFigure 3-2. Calibration plot of random effects model**

**
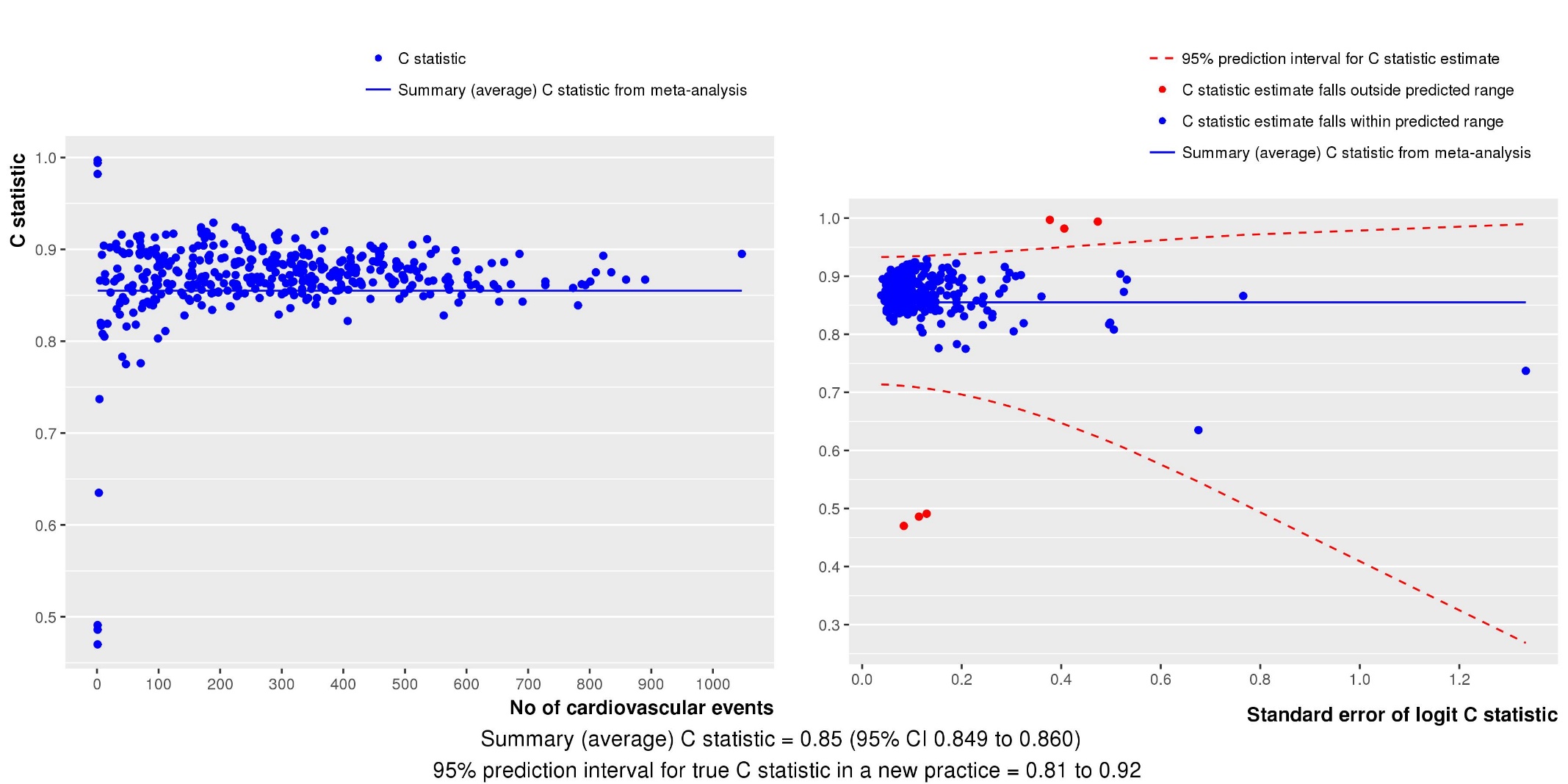
**

**eFigure 4**. Variation of QRISK3’s C-statistic among practices—

a replication of Riley’s^1^ funnel plot
